# A global analysis of the value of precision medicine in oncology – The case of non-small cell lung cancer

**DOI:** 10.3389/fmed.2023.1119506

**Published:** 2023-02-20

**Authors:** Thomas Hofmarcher, Chiara Malmberg, Peter Lindgren

**Affiliations:** ^1^IHE–The Swedish Institute for Health Economics, Lund, Sweden; ^2^Karolinska Institutet, Solna, Sweden

**Keywords:** precision medicine, personalized medicine, molecular diagnostics, companion diagnostic testing, cost-effectiveness, survival, non-small cell lung cancer, partitioned survival model

## Abstract

**Objectives:**

Biomarker testing is indispensable for the implementation of precision medicine (PM) in oncology. The aim of this study was to assess the value of biomarker testing from a holistic perspective based on the example of advanced non-small cell lung cancer (aNSCLC).

**Materials and methods:**

A partitioned survival model was populated with data from pivotal clinical trials of first-line treatments in aNSCLC. Three testing scenarios were considered; “no biomarker testing” encompassing chemotherapy treatment, “sequential testing” for EGFR and ALK encompassing treatment with targeted- or chemotherapy, and “multigene testing” covering EGFR, ALK, ROS1, BRAF, NTRK, MET, RET and encompassing treatment with targeted- or immuno(chemo)therapy. Analyses of health outcomes and costs were run for nine countries (Australia, Brazil, China, Germany, Japan, Poland, South Africa, Turkey, United States). A 1-year and 5-year time horizon was applied. Information on test accuracy was combined with country-specific information on epidemiology and unit costs.

**Results:**

Compared to the no-testing scenario, survival improved and treatment-related adverse events decreased with increased testing. Five-year survival increased from 2% to 5–7% and to 13–19% with sequential testing and multigene testing, respectively. The highest survival gains were observed in East Asia due to a higher local prevalence of targetable mutations. Overall costs increased with increased testing in all countries. Although costs for testing and medicines increased, costs for treatment of adverse events and end-of-life care decreased throughout all years. Non-health care costs (sick leave and disability pension payments) decreased during the first year but increased over a 5-year horizon.

**Conclusion:**

The broad use of biomarker testing and PM in aNSCLC leads to more efficient treatment assignment and improves health outcomes for patients globally, in particular prolonged progression-free disease phase and overall survival. These health gains require investment in biomarker testing and medicines. While costs for testing and medicines would initially increase, cost decreases for other medical services and non-health care costs may partly offset the cost increases.

## 1. Introduction

Biomarker testing has become indispensable for the implementation of precision medicine (PM) in oncology (1, 2). Traditional therapeutic regimens applied the same regimen to patients with the same disease, such as chemotherapy to all patients with advanced non-small cell lung cancer (aNSCLC) (3). With PM, patients undergo prior testing to identify molecular targets which enables patient stratification and biomarker-driven therapeutic regimens, such as epidermal growth factor receptor (EGFR)-targeted therapy to patients with an EGFR mutation (3). This is supposed to increase the efficiency of care delivery by reducing the administration of treatments with uncertain effectiveness. PM is also supposed to improve health outcomes and may reduce treatment-related toxicities (4).

On the cost side, PM affects health care costs in various ways (5). The administration of PM necessitates infrastructure for testing and the testing needs to be done in the whole patient population, which increases costs compared to no testing. Medicine costs might increase if newer on-patent medicines replace off-patent medicines but might also decrease by preventing the prescription of treatments to patients who do not benefit from them. Other health care costs for repeated treatment attempts after failure of initial treatment as well as hospital admissions for treatment-related adverse events might decrease. However, a recent review of studies on the health care costs and benefits of PM in oncology concluded that there is limited evidence of PM being cost-effective (6). Yet this review neglected the wider, societal impact of PM that encompasses additional elements besides health care costs and health outcomes (7). For example, non-health care costs arising from sick leave and early retirement of working-age patients might decrease as patients’ health outcomes improve (8).

Lung cancer is the second most diagnosed cancer type and the most common cause of cancer-related death globally, with an estimated 2.2 million new cases and 1.8 million deaths in 2020 (9). The overall prognosis is poor, with 5-year relative survival rates of lung cancer mostly being between 10 and 21% in Europe and Northern America and up to 30% in East Asia in patients diagnosed in 2010–2014 (10). NSCLC accounts for around 85% of all lung cancer cases and more than half of all new NSCLC cases are diagnosed at an advanced stage (11).

The poor outcomes of lung cancer patients indicate a large need for improved diagnostic and treatment approaches. Over the past two decades, the development of molecular profiling and targeted therapeutic agents has heralded the introduction of PM, particularly in aNSCLC (12–14). New treatments involve biomarkers that target various receptors, pathways, and proteins. However, the administration of new treatments requires prior biomarker testing, with an increasing need to switch from upfront single biomarker testing to multigene testing.

The aim of this study was to assess the value of biomarker testing–in terms of health improvements and cost changes–for the implementation of PM in oncology based on the example of aNSCLC. A holistic perspective, including the impact on patient health, the health system, and the treasury (i.e., public social security payments), was applied. High-income and middle-income countries representative of different regions of the world were analyzed and different scenarios reflecting past and future biomarker testing were used.

## 2. Materials and methods

A health economic model was developed for the treatment of aNSCLC and a health care and treasury perspective applied. Systemic therapy is the standard treatment in aNSCLC, according to guidelines from the American National Comprehensive Cancer Network (NCCN) and the European Society for Medical Oncology (ESMO) (15, 16). Three different scenarios for upfront biomarker testing and ensuing first-line systemic therapy in aNSCLC were considered. Health-related and cost-related outcomes were modeled and compared between these scenarios.

Analyses were run for nine high-income and middle-income countries across all continents, Australia, Brazil, China, Germany, Japan, Poland, South Africa, Turkey, and the United States. The choice of countries was informed by data availability, population size, and representativeness for similar countries in their respective region. No low-income countries were included because it seemed unrealistic to assume the wide adoption of PM within the next 10 years in these countries.

### 2.1. Scenarios

**FIGURE 1:**
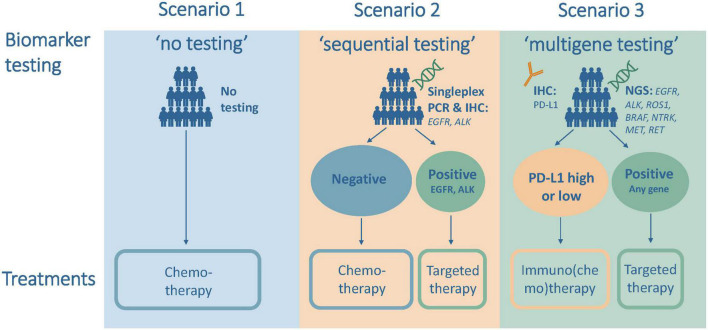
Testing and treatment scenarios for the analysis of first-line treatment of advanced non-small cell lung cancer (aNSCLC).

Three testing scenarios were defined in the model to illustrate the gradual introduction of biomarker testing followed by the administration of matching systemic therapies in the first-line treatment of aNSCLC; see Figure 1. The first scenario was “no biomarker testing,” which did not include any upfront biomarker testing and led to the administration of chemotherapy in all patients. This scenario modeled the situation in high-income countries in the mid-2000s.

The second scenario was “sequential testing,” which included upfront sequential and single biomarker testing for EGFR and anaplastic lymphoma kinase (ALK) followed by treatment with targeted therapy for mutation-positive patients and with chemotherapy for all other patients. This scenario modeled the situation in high-income countries just before the introduction of immunotherapy in 2015 and the current situation in many middle-income countries.

The third scenario was “multigene testing,” which included upfront next-generation sequencing (NGS) testing and treatment with targeted therapy if tested positive for the presence of EGFR, ALK, ROS proto-oncogene 1 (ROS1), B-Raf proto-oncogene (BRAF), neurotrophic tyrosine receptor kinase (NTRK), mesenchymal epithelial transition (MET), or ret proto-oncogene (RET), and testing for PD-L1 status and treatment with immuno(chemo)therapy for all other patients. This scenario modeled the situation in 2022 or the near future in most high-income countries.

### 2.2. Outcome measures

The model included four main outcome measures per patient. The first measure was survival, measured as both the number of life years after treatment start (separated in the model into years spent progression-free and in progression after first-line therapy) and the absolute survival rate as the proportion of patients alive after a certain number of years. The second measure was the number of treatment-related adverse events (AEs), which served as a proxy measure for quality of life. The third measure was health care costs, calculated as the sum of costs for tests, medicines, administration of medicines, treatment of treatment-related AEs, other medical services, and end-of-life care. The fourth measure was non-health care costs from a treasury perspective, calculated as the sum of public social security payments for temporary sick leave and disability pension due to early retirement. Productivity losses were not considered.

A 1-year and 5-year time horizon was applied in the measurement of all outcomes. The first year after diagnosis was supposed to capture the period when patients receive first-line therapy, while the 5-year cutoff is the standard length in analyses of long-term survival (10).

### 2.3. Model structure

A partitioned survival model with three health states–progression-free, progressed, and dead–and a monthly cycle length was developed (17). Information on overall survival (OS), describing the time from model entry (i.e., treatment start) to death, and progression-free survival (PFS), describing the time from model entry to exiting the progression-free state *via* progression or death, were included for each first-line systemic therapy in aNSCLC. Kaplan-Meier curves for the therapies were sourced from publications of pivotal clinical trials and then digitalized. Survival functions for the OS and PFS curves were estimated using a Weibull distribution for all therapies. Health effects and costs were attached to each state and therapy.

### 2.4. Model inputs

At the start of the analysis, a biopsy was assumed to be performed before the administration of first-line therapies. In the first scenario, no additional biomarker testing was performed. In the second scenario, all patients were first tested for EGFR mutations using a singleplex polymerase chain reaction (PCR) test, and if negative for ALK mutations using an immunohistochemistry assay. In the third scenario, all patients were tested for the presence of all included mutations simultaneously using an NGS test as well as a separate PD-L1 test. Country-specific prevalences of mutations and gene expressions in NSCLC and information on the accuracy of the tests (sensitivity and specificity) were obtained from public sources; see Supplementary Tables 1, 2. Unit costs for the tests were obtained from a recent publication for the United States (18); see Supplementary Table 6. These costs were converted using differences in purchasing power parities (PPP) to estimate local costs in the remaining countries.

A total of 25 first-line systemic therapies in aNSCLC were included in the scenarios. This comprised first-line therapies with an approval by the United States Food and Drug Administration (US FDA) until December 31, 2021; see Supplementary Table 3. For each therapy, the approved dosage (standardized to milligrams per month), the administration frequency, and the approved length of administration were obtained from public sources; see Supplementary Table 4. Medicine costs, based on list prices of medicine packs and standardized to costs per milligram, were obtained for each country from Eversana; see Supplementary Table 7. Country-specific unit costs for an intravenous administration were obtained from public sources (see Supplementary Table 7), whereas no administration costs were assumed for oral medicines. In the model, medicines were being administered as long as patients were in the progression-free state unless the maximum number of administration cycles had been reached. In the progressed state, all patients were assumed to receive 3 months of intravenously administered chemotherapy irrespective of the type of previous treatment and were applied as one-time costs. This assumption was based on the approximate treatment length observed in the control arms in pivotal clinical trials of immunotherapies for second-line treatment. Costs in the first cycle of progression were applied to all progressed patients, while in later cycles, costs were only applied to the additional patients in the progressed state, in order to avoid double counting. No later line treatments were included.

The occurrence of treatment-related AEs for all included therapies was sourced from publications of pivotal clinical trials based on the reported frequency irrespective of grade; see Supplementary Table 5. Based on input from an external clinical adviser, the following eight common AEs were included in the model; anemia, neutropenia, fatigue, nausea, vomiting, diarrhea, aspartate amino transferase (ASAT) increase, alanine amino transferase (ALAT) increase. Treatment-related AEs were assumed to only occur as long as systemic therapy was being administered in both the progression-free and the progressed state. Treatment costs for each AE were obtained from public sources for the United States and converted to local costs in the remaining countries using differences in PPP; see Supplementary Table 6.

The model also included other monthly medical service costs reflecting outpatient visits, interventions, and unplanned hospitalizations unrelated to AEs from a previous publication for the United States (19). These costs were converted to local costs in the remaining countries using differences in PPP. The costs varied by broad type of systemic therapy (chemotherapy, immunotherapy, targeted therapy); see Supplementary Table 6. The same monthly costs were assumed for the entire time spent in the progression-free state and the progressed state.

The dead state in the model included a one-time cost for end-of-life care (i.e., palliative care). Unit costs for 1 month of end-of-life care were obtained from public sources for each country; see Supplementary Table 7.

Non-health care costs included public payments for temporary sick leave during the progression-free state and for disability pension in the progressed state. Only patients of working age [defined to range from 18 years to the official retirement age for men and women in each country (20)] who could be expected to have been employed at the time of diagnosis [approximated by the sex-specific employment rate in each country (21)] were assumed to receive these payments. The proportion of patients in working age was derived from age-specific lung cancer incidence numbers (assumed to be representative for aNSCLC) for all countries from the Global Cancer Observatory (22). In the progression-free state, only patients not returning to work were assumed to receive sick leave payments, assuming a uniform return-to-work proportion in all countries based on an international review study (23). Monthly sick leave payments and disability pension payments were calculated based on country-specific information mainly coming from the International Labour Organization and the International Social Security Association (20, 24); see Supplementary Table 7.

As the model horizon covered up to 5 years, future health outcomes and costs were discounted with a 3% annual discount rate. All costs were expressed in US-dollars ($) per patient in 2021 prices and exchange rates using information from the World Bank (21).

## 3. Results

The traditional treatment regimen in aNSCLC involved no biomarker testing and the administration of chemotherapy to all patients. The introduction of biomarker testing and targeted therapies and immunotherapy has led to a complete overhaul of the treatment regimen. The results of the analysis illustrate the effects of the gradual introduction of biomarker testing and PM in two scenarios of sequential testing or multigene testing compared to a no-testing scenario. The effects are shown for both a 1-year and a 5-year time horizon after treatment start.

**FIGURE 2:**
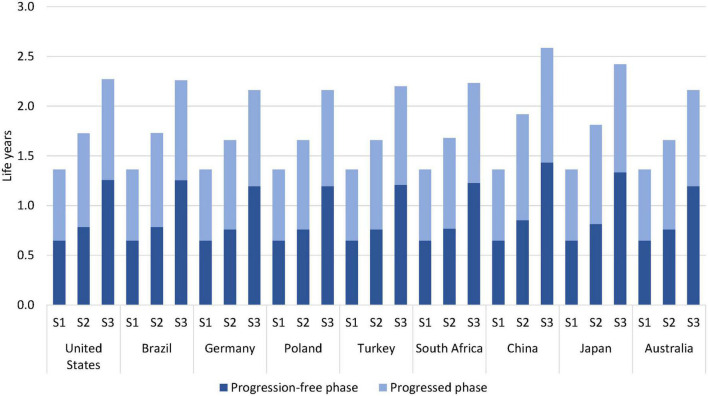
Estimated life years per patient by testing scenario over a 5-year horizon. S1 = no testing, S2 = sequential testing, S3 = multigene testing.

The results showed that survival of patients improved with sequential testing and multigene testing compared to no testing; see Table 1. The mean remaining life years after treatment start increased from around 1.4 years with no testing to 1.7–1.9 years with sequential testing and 2.2–2.6 years with multigene testing over the full model horizon. The increase with multigene testing was driven by almost a doubling of the progression-free disease phase and by a smaller increase in the progressed phase; see Figure 2. The 1-year survival rate increased from 51 to 60–68% and 71–80% with sequential and multigene testing, respectively, and the 5-year survival rate increased from 2 to 5–7% and 13–19%. The biggest improvements were observed in China and Japan, because of the higher local prevalence of targetable mutations (in particular EGFR) and availability of effective treatments.

**TABLE 1:** Health-related outcome measures per patient.

	1-year			5-year		
	No testing	Sequential testing	Multigene testing	No testing	Sequential testing	Multigene testing
**Life years**						
United States	0.82	0.88	0.96	1.36	1.73	2.27
Brazil	0.82	0.89	0.96	1.36	1.73	2.26
Germany	0.82	0.87	0.94	1.36	1.66	2.16
Poland	0.82	0.87	0.94	1.36	1.66	2.16
Turkey	0.82	0.87	0.94	1.36	1.66	2.20
South Africa	0.82	0.88	0.95	1.36	1.68	2.23
China	0.82	0.92	1.00	1.36	1.92	2.59
Japan	0.82	0.90	0.97	1.36	1.81	2.42
Australia	0.82	0.87	0.94	1.36	1.66	2.16
**Absolute survival rate**						
United States	51%	62%	73%	2%	6%	15%
Brazil	51%	62%	73%	2%	6%	14%
Germany	51%	60%	71%	2%	5%	13%
Poland	51%	60%	71%	2%	5%	13%
Turkey	51%	60%	71%	2%	5%	14%
South Africa	51%	60%	72%	2%	5%	15%
China	51%	68%	80%	2%	7%	19%
Japan	51%	64%	76%	2%	6%	17%
Australia	51%	60%	71%	2%	5%	13%
**Number of adverse events**						
United States	1.10	0.90	0.42	1.21	1.11	0.87
Brazil	1.10	0.90	0.42	1.21	1.11	0.87
Germany	1.10	0.94	0.44	1.21	1.14	0.84
Poland	1.10	0.94	0.44	1.21	1.14	0.84
Turkey	1.10	0.94	0.43	1.21	1.14	0.84
South Africa	1.10	0.93	0.42	1.21	1.13	0.85
China	1.10	0.77	0.35	1.21	1.02	0.98
Japan	1.10	0.84	0.39	1.21	1.07	0.92
Australia	1.10	0.94	0.44	1.21	1.14	0.84

**FIGURE 3:**
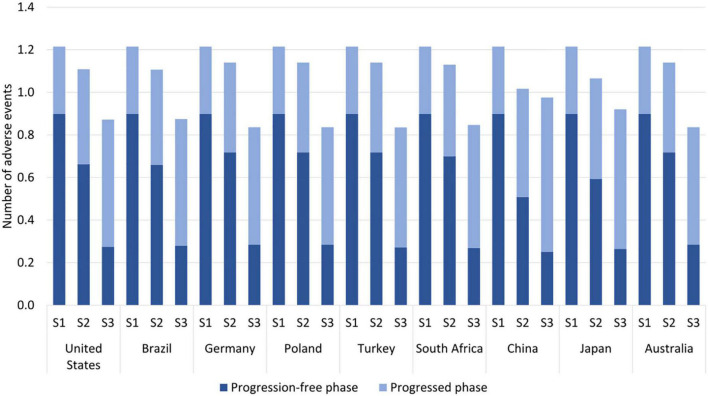
Estimated number of adverse events per patient by testing scenario over a 5-year horizon. S1 = no testing, S2 = sequential testing, S3 = multigene testing. The following eight adverse events (AEs) were included; anemia, neutropenia, fatigue, nausea, vomiting, diarrhea, aspartate amino transferase (ASAT) increase, alanine amino transferase (ALAT) increase.

The overall number of treatment-related AEs decreased with sequential and multigene testing; see Table 1 and Figure 3. This decrease occurred despite longer administration times of targeted medicines and immuno(chemo)therapy medicines than of chemotherapy in the no-testing scenario. The decrease was especially pronounced during the first year of treatment (reduction of 64–72% with multigene testing) when patients typically receive PM-based first-line therapy. A somewhat smaller decrease was observed in China and Japan over a 5-year horizon, driven by the AE-profile of EGFR medicines and the higher prevalence of EGFR mutations.

**FIGURE 4:**
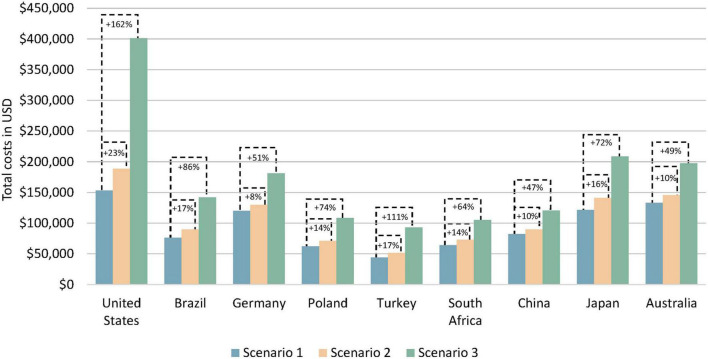
Estimated total costs per patient by testing scenario over a 5-year horizon. Scenario 1 = no testing, scenario 2 = sequential testing, scenario 3 = multigene testing. Total costs are the sum of health care costs and non-health care costs.

The total costs as the sum of health care and non-health care costs with sequential testing compared to no testing remained mostly unchanged (ranging from a 22% decrease in China to a 7% increase in the United States) during the first year of treatment; see Table 2. With multigene testing, the change in total costs compared to no testing ranged from a 14% decrease in China to a 117% increase in the United States. Over the 5-year horizon, total costs increased slightly with sequential testing compared to no testing in all countries, ranging from an 8% increase in Germany to a 23% increase in the United States; see Figure 4. The increases were larger with multigene testing ranging from a 47% increase in China to a 162% increase in the United States. The total per-patient costs with multigene testing were close to $100,000 in China, Poland, South Africa, and Turkey, around $140,000 in Brazil, close to $200,000 in Australia, Germany, and Japan, and $400,000 in the United States. Non-health care costs were small in comparison with health care costs in all countries.

**TABLE 2:** Overall cost-related outcome measures per patient (all in USD).

	1-year			5-year		
	No testing	Sequential testing	Multigene testing	No testing	Sequential testing	Multigene testing
**Total costs**						
United States	100,695	107,412	218,389	153,044	188,582	401,313
Brazil	48,812	47,750	66,950	76,318	89,673	141,976
Germany	77,258	71,809	85,356	120,032	129,917	181,102
Poland	39,474	38,812	51,860	62,416	71,071	108,660
Turkey	28,049	28,589	46,622	44,144	51,628	92,993
South Africa	40,890	39,831	48,657	64,069	72,892	105,271
China	51,361	40,308	44,370	82,140	89,953	120,388
Japan	76,654	70,610	87,878	121,696	141,205	208,944
Australia	83,918	78,387	88,656	133,071	145,892	197,746
**Health care costs**						
United States	100,025	106,721	217,951	151,075	185,983	398,517
Brazil	48,218	47,123	66,419	74,926	87,855	139,898
Germany	74,296	68,617	81,539	116,218	125,386	174,456
Poland	38,817	38,109	51,071	61,411	69,858	107,019
Turkey	27,722	28,237	46,211	43,690	51,086	92,213
South Africa	40,720	39,649	48,454	63,803	72,566	104,825
China	50,843	39,724	43,750	81,254	88,703	118,722
Japan	75,526	69,373	86,618	119,634	138,438	205,388
Australia	83,060	77,470	87,632	131,741	144,283	195,583
**Non-health care costs**						
United States	669	691	438	1,969	2,599	2,796
Brazil	594	627	531	1,393	1,817	2,078
Germany	2,962	3,192	3,817	3,813	4,531	6,646
Poland	657	703	789	1,004	1,213	1,641
Turkey	328	352	411	453	543	780
South Africa	170	182	203	266	327	445
China	518	584	620	886	1,250	1,667
Japan	1,128	1,237	1,260	2,063	2,767	3,556
Australia	858	917	1,024	1,330	1,609	2,163

Although the total costs increased with sequential and multigene testing in all countries over a 5-year horizon, the different types of health care and non-health care costs included in the model exhibited different trajectories; see Table 3. Costs for biomarker testing increased naturally compared to the no testing scenario which only included costs for a biopsy.

**TABLE 3:** Detailed cost-related outcome measures per patient (all in USD).

	1-year			5-year		
	No testing	Sequential testing	Multigene testing	No testing	Sequential testing	Multigene testing
**Test costs**						
United States	552	1,803	5,227	552	1,803	5,227
Brazil	342	913	2,483	342	913	2,483
Germany	110	1,229	4,053	110	1,229	4,053
Poland	317	926	2,463	317	926	2,463
Turkey	80	496	1,545	80	496	1,545
South Africa	317	868	2,296	317	868	2,296
China	292	920	3,126	292	920	3,126
Japan	383	1,475	4,815	383	1,475	4,815
Australia	151	1,471	4,803	151	1,471	4,803
**Medicine costs**						
United States	16,561	36,633	159,576	18,355	48,131	247,237
Brazil	1,782	8,107	34,315	1,942	11,364	55,954
Germany	2,131	5,539	27,964	2,336	7,618	45,327
Poland	599	4,442	23,061	662	6,447	38,079
Turkey	958	4,703	26,685	1,059	6,636	43,082
South Africa	1,884	5,935	20,660	2,085	8,115	33,489
China	530	2,204	13,444	582	3,104	21,173
Japan	2,247	10,553	38,169	2,480	14,723	65,666
Australia	151	4,354	27,069	166	6,372	45,146
**Medicine administration costs**						
United States	3,070	2,060	2,757	3,369	2,263	3,635
Brazil	1,195	798	1,066	1,312	876	1,394
Germany	4,026	2,980	4,124	4,418	3,275	5,411
Poland	602	445	616	660	490	809
Turkey	496	367	507	544	403	677
South Africa	605	434	594	664	477	794
China	17	8	10	19	9	13
Japan	139	81	108	152	89	145
Australia	770	570	789	845	626	1,035
**Adverse events costs**						
United States	10,480	8,261	3,514	11,537	10,135	7,594
Brazil	6,384	5,018	2,161	7,028	6,163	4,643
Germany	9,102	7,571	3,246	10,020	9,098	6,310
Poland	5,073	4,220	1,809	5,585	5,071	3,517
Turkey	3,543	2,948	1,227	3,901	3,542	2,445
South Africa	5,166	4,226	1,756	5,687	5,111	3,616
China	6,698	4,463	1,860	7,373	5,860	5,464
Japan	9,885	7,256	3,059	10,882	9,145	7,584
Australia	11,512	9,577	4,105	12,673	11,507	7,980
**Other medical costs**						
United States	62,324	52,396	42,973	103,841	110,818	123,321
Brazil	37,972	31,859	26,092	63,267	67,549	74,531
Germany	54,130	47,296	39,256	90,189	95,363	105,366
Poland	30,171	26,362	21,880	50,269	53,153	58,728
Turkey	21,074	18,413	15,310	35,112	37,126	41,878
South Africa	30,726	26,525	21,973	51,194	54,289	61,320
China	39,836	29,804	23,857	66,372	72,596	83,589
Japan	58,788	46,998	38,449	97,950	105,615	120,684
Australia	68,470	59,826	49,656	114,082	120,626	133,279
**End-of-life care costs**						
United States	7,039	5,568	3,904	13,420	12,834	11,503
Brazil	544	429	302	1,036	991	892
Germany	4,796	4,001	2,897	9,145	8,803	7,990
Poland	2,055	1,714	1,241	3,918	3,772	3,423
Turkey	1,571	1,310	937	2,995	2,883	2,585
South Africa	2,023	1,660	1,175	3,857	3,705	3,311
China	3,470	2,326	1,453	6,617	6,215	5,356
Japan	4,084	3,010	2,017	7,787	7,391	6,493
Australia	2,005	1,673	1,211	3,824	3,681	3,341
**Sick leave payments**						
United States	0	0	0	0	0	0
Brazil	196	216	272	221	267	427
Germany	2,702	2,927	3,638	3,050	3,570	5,612
Poland	512	554	689	578	676	1,063
Turkey	282	305	379	318	372	592
South Africa	129	140	175	145	172	275
China	352	408	528	397	523	876
Japan	693	781	996	782	982	1,608
Australia	659	713	887	743	870	1,368
**Disability pension payments**						
United States	669	691	438	1,969	2,599	2,796
Brazil	398	411	259	1,172	1,550	1,651
Germany	260	265	179	764	961	1,035
Poland	145	148	100	427	537	578
Turkey	46	47	32	136	171	188
South Africa	41	42	28	121	155	171
China	166	176	91	489	727	790
Japan	435	455	263	1,281	1,785	1,948
Australia	200	204	138	587	739	796

Medicine costs increased around 2–10-fold in most countries with sequential testing compared to no testing over a 5-year horizon. With multigene testing, medicine costs increased mostly by a factor of 14–58 compared to no testing. These large relative increases were primarily driven by older and low-cost off-patent chemotherapies being replaced with newer on-patent targeted therapies and immunotherapies. The longer administration time of newer medicines (often until disease progression or unacceptable toxicity) compared to chemotherapy (often a fixed number of treatment cycles) also contributed to the cost increase.

Medicine administration costs decreased by up to 50% with sequential testing. This was because patients with targetable mutations switched from receiving intravenously administered medicines to oral medicines for which no administration costs arose. Administration costs with multigene testing over a 5-year horizon were higher than with no testing and sequential testing (except in China and Japan) despite a higher proportion of patients receiving oral targeted therapies. This was caused by patients without targetable mutations receiving intravenous immuno(chemo)therapy for a longer time than only chemotherapy.

Costs of treatment-related AEs decreased with sequential and multigene testing. The decrease was particularly strong (64–72% decrease) in the first year with multigene testing. This mirrors the development seen in Table 1 for the occurrence of AEs. Most patients in the no-testing scenario already progressed on their first-line therapy and started receiving second-line chemotherapy with associated AEs during the first year. By contrast, patients in the multigene testing scenario mostly received their PM-based first-line therapy during the first year. Costs of treatment-related AEs with multigene testing rise after the first year, driven both by the continued treatment with first-line therapy and by the eventual administration of chemotherapy as a second-line therapy, but sustain a cost reduction of 26–37% over 5 years in all countries.

Other medical service costs for outpatient visits, interventions, and unplanned hospitalizations unrelated to AEs decreased with sequential testing (13–25%) and multigene testing (27–40%) in the first year but increased over the 5-year horizon (6–9 and 17–26%, respectively) in all countries. The initial decrease was driven by lower monthly costs for these services in patients receiving targeted therapy and immuno(chemo)therapy. The subsequent increase was driven by the prolonged survival of patients with sequential and multigene testing.

End-of-life care costs decreased in all countries by up to 33% with sequential testing and 58% with multigene testing in the first year, mirroring the increase in the absolute survival rate shown in Table 1. Over time, the cost reduction became smaller (4–6 and 13–19%, respectively) but was sustained over the 5-year horizon.

The two types of non-health care costs were affected in different ways by increased biomarker testing. With sequential and multigene testing, sick leave payments increased already during the first year and continued to increase over the 5-year horizon (17–32 and 84–121%, respectively) in all countries (except in the United States with no such payments), driven by prolonged survival. Disability pension payments decreased by 31–45% during the first year in all countries with multigene testing due to the prolonged progression-free disease phase. Yet these payments increased over the 5-year horizon with multigene testing (36–62%) as more patients maintained a survival advantage compared to the no-testing scenario.

To confirm the robustness of the main results, a sensitivity analysis of the accuracy (i.e., sensitivity and specificity) of the biomarker tests in all testing scenarios was carried out; see Supplementary Table 8. As expected, there were no changes in the no testing scenario. With sequential testing and multigene testing, the differences in life years and total costs compared to the base case values were generally small.

## 4. Discussion

The treatment possibilities of aNSCLC have changed radically with the introduction of numerous targeted therapies and immuno(chemo)therapy. The administration of these treatments requires a change from single biomarker testing to multigene testing. Multigene testing avoids overly long delays in treatment start and enables the administration of PM as initial therapy.

The results in this study showed that multigene testing followed by the administration of matching PM improved various health outcomes compared to no testing or sequential testing in all countries. This included an improved survival rate, an increased number of life years, a prolonged progression-free disease phase, and a reduced number of treatment-related AEs. The largest improvements in life years were observed in East Asian countries due to a higher prevalence of targetable mutations in the local patient population. Multigene testing also improved treatment assignment (for patients with EGFR and ALK mutations) because the sensitivity and specificity of NGS testing was higher than the sensitivity/specificity of the single biomarker tests in the sequential testing scenario.

For all countries, total costs increased with sequential testing and multigene testing compared to no testing, yet this development was not shared by all individual cost items. The testing costs for multigene testing were higher than for sequential testing, but the increase in testing costs was dwarfed by the increase in medicine costs. The latter was partly the result of on-patent medicines replacing off-patent chemotherapy medicines in all countries. The replacement of intravenously administered chemotherapy with oral agents reduced medicine administration costs in some countries. Health care costs for the treatment of AEs and end-of-life care also decreased with multigene testing. Non-health care costs decreased initially with multigene testing in some countries thanks to a prolonged progression-free disease phase but increased over time in line with overall survival.

Clinical guidelines from the NCCN, ESMO, the College of American Pathologists, the International Association for the Study of Lung Cancer, and the Association for Molecular Pathology recommend molecular testing in aNSCLC when feasible and to be performed with NGS (15, 25, 26). Europe’s Beating Cancer Plan also includes the aim to use NGS testing in cancer patients more broadly (27). However, there may be a substantial disconnect between recommendations in clinical and political guidelines and actual clinical practice (1). In various countries in Europe, previous studies have shown that many patients with aNSCLC cannot access NGS testing (13, 28). This is partly a result of non-reimbursement of NGS testing by the public health care payers and partly of a lack of laboratory infrastructure and qualified workforce to perform the testing. There are also examples of public payers reimbursing newer targeted medicines (e.g., for ROS1, BRAF, and NTRK) but not NGS testing, effectively limiting patient access to these medicines as first-line treatments, as sequential single biomarker testing would take too long to be realistic option (13).

The value of NGS testing in aNSCLC is increasing with the identification of additional oncogenic drivers and the development of matching targeted therapies. The third scenario in the model included seven targets besides PD-L1, based on all US FDA approved first-line medicines. Additional targets that may become relevant in the coming years are KRAS, HER2, and PIK3CA (29–31). For KRAS G12C and HER2 mutations, the US FDA already approved new medicines as a second-line therapy in 2021 and 2022, respectively, but their future use in first line as a monotherapy might be limited, unless they can be shown to be safe and more effective than the current standard of immuno(chemo)therapy (32, 33). However, their use in combination with immunotherapy might become possible in the coming years (34).

Multigene testing is also set to become increasingly important in earlier stages of NSCLC for the adjuvant treatment after surgery. In 2020, the US FDA approved the first EGFR-targeted medicine in this setting (35), and results from a pivotal trial of an ALK-targeted medicine are expected in 2023 (36). Other targets might follow. Broad biomarker testing in all newly diagnosed NSCLC cases irrespective of disease stage thus might become standard clinical practice in the coming years. The cost implications of NGS testing in the adjuvant setting might be largely similar to the metastatic setting, except for non-health care costs. Public payments for temporary sick leave and disability pension might see greater and sustained reductions over time due to much longer periods of disease-free survival, as observed with EGFR-directed treatment (37).

### 4.1. Limitations

As a partitioned survival model with three states was used, the assignment of patients in these states at different points in time by treatment is critical. The Kaplan-Meier curves of treatments used for this purpose typically had fewer than 5 years of follow-up. Weibull distributions were used to estimate all survival functions for the full 5-year horizon of the model, but these survival functions go to zero with increasing time. The functions do not capture survival plateaus that are indicative of cure well, leading to an underestimation of the true size of the survival outcomes. In addition, the model feeds on survival data from different clinical trials with somewhat heterogenous patient populations due to varying inclusion criteria across clinical trials.

The analysis assumed that all patients are tested and would receive appropriate treatment corresponding to their test results. In clinical practice, not every patient with aNSCLC gets tested and not every tested patient will receive the appropriate medicine. A recent analysis in European countries indicated that the use of targeted therapy and immuno(chemo)therapy was below the level recommended in clinical guidelines (38).

Medicine costs were the second largest cost item in the scenario with multigene testing in most countries. It is important to reiterate that the analysis drew on country-specific list prices of all medicines in the absence of publicly available net prices. List prices may deviate from net prices due to confidential rebates granted by pharmaceutical companies to the payer. For example, an analysis of branded prescription medicines in the United States showed that the average size of rebates increased markedly over the last decade, reaching over 50% of the list prices in 2019 (39). The medicine costs in the scenarios for sequential testing (where some medicines were branded medicines) and especially for multigene testing (where nearly all medicines were branded medicines) might therefore be greatly overestimated in this study.

The estimation of medicine costs for subsequent treatment lines in the progression state was approximated by assigning costs to all additional patients in the progressed health state compared to the previous cycle. This may have underestimated the medicine costs in the progressed state, as the difference did not take into account patients who had died in the previous cycle.

Unit costs for biomarker tests, treatment of AEs, and other medical services were only obtained for the United States. The conversion of these costs to other countries took into account differences in PPP, but this should still be considered a crude conversion. The true costs of these three cost items in other countries could thus be either overestimated or underestimated. The unit costs of NGS testing were sourced from a recent publication for the year 2020 (18). These costs might have decreased since then and continue to decrease in the future with increasing economies of scale and as technology and experience improves. Indeed, a decreasing price development of NGS tests in 2016–2019 has been observed in the United States (40).

## 5. Conclusion

The use of PM in oncology is set to increase in the coming years thanks to advances in the understanding of molecular mechanisms and expressions involved in the growth of cancer tumors. A prime example is aNSCLC, where the number targetable mutations have already multiplied during the last decade. This development has created a need to improve biomarker testing, moving away from upfront single biomarker testing to multigene testing. Other cancer types might face similar circumstances in the near future.

The example in this study illustrates that the gradual introduction of PM in aNSCLC improves health outcomes in countries around the world, with the largest improvements in survival observed in East Asia due to a higher prevalence of targetable mutations in the local patient population. Although the use of PM in aNSCLC leads to increased costs for biomarker testing and medicines in all countries, other health care costs are decreased, notably for the treatment of AEs and end-of-life care. Testing costs are also minimal in relation to medicine costs. Non-health care costs decrease in the first year after treatment start in some countries but increase subsequently. The overall results demonstrate the importance to apply a wider perspective in the assessment of the value of PM in oncology.

## Data availability statement

The original contributions presented in this study are included in the article/Supplementary material, further inquiries can be directed to the corresponding author.

## Author contributions

TH and PL: conceptualization. TH and CM: data analysis and writing—original draft. All authors contributed to the data interpretation, writing—review and editing, and approved the submitted version.
