# Supplementary material for: A global analysis of the value of precision medicine in oncology – The case of non-small cell lung cancer
Source: Front Med (Lausanne). 2023 Feb 20;10:1119506. doi: 10.3389/fmed.2023.1119506 (PMC9986274; doi:10.3389/fmed.2023.1119506)
Supplement: Supplementary file 1 [file Table_1.DOCX]

Supplementary Material

A Global Analysis of the Value of Precision Medicine in Oncology – the Case of Non-Small Cell Lung Cancer

Thomas Hofmarcher*, Chiara Malmberg, Peter Lindgren

*** Correspondence:** Corresponding Author: thomas.hofmarcher@ihe.se

# Supplementary Tables

Table S1. Prevalence of mutations and gene expressions by country.

|  | United States | Brazil | Germany | Poland | Turkey | South Africa | China | Japan | Australia | Source |
| --- | --- | --- | --- | --- | --- | --- | --- | --- | --- | --- |
| PD-L1 high* | 25.0% | 20.0% | 25.0% | 25.0% | 35.0% | 35.0% | 30.0% | 30.0% | 25.0% | (1) |
| PD-L1 non-high* | 75.0% | 80.0% | 75.0% | 75.0% | 65.0% | 65.0% | 70.0% | 70.0% | 75.0% | (1) |
| EGFR | 23.9% | 24.4% | 14.1% | 14.1% | 14.1% | 17.2% | 51.7% | 36.6% | 14.1% | (2, 3) |
| ALK | 4.5% | 4.5% | 4.5% | 4.5% | 4.5% | 4.5% | 3.9% | 3.9% | 4.5% | (2, 4) |
| ROS1 | 1.5% | 1.5% | 1.5% | 1.5% | 1.5% | 1.5% | 0.9% | 0.9% | 1.5% | (2, 5) |
| BRAF | 1.3% | 1.3% | 1.3% | 1.3% | 1.3% | 1.3% | 1.0% | 1.0% | 1.3% | (2, 6, 7) |
| NTRK | 0.2% | 0.2% | 0.2% | 0.2% | 0.2% | 0.2% | 0.6% | 0.6% | 0.2% | (2, 8) |
| RET | 1.5% | 1.5% | 1.5% | 1.5% | 1.5% | 1.5% | 1.4% | 1.4% | 1.5% | (2, 9) |
| MET | 3.0% | 3.0% | 3.0% | 3.0% | 3.0% | 3.0% | 0.5% | 0.5% | 3.0% | (2, 10) |

Notes: The PD-L1 ‘high’ refers to a ≥50% expression of tumor proportion score and ‘non-high’ to <50% expression. ALK refers to rearrangement-positive cases. BRAF refers to cases with a BRAF V600E mutation. MET refers to cases with exon 14 skipping. * The proportions of PD-L1 high and non-high were resized to the size of the population without any included driver mutations in the scenario with multigene testing.

Table S2. Biomarker tests specification.

| Type of test | Test name | Sensitivity | Specificity | Source |
| --- | --- | --- | --- | --- |
| Singleplex PCR – EGFR | Therascreen EGFR RGQ PCR Kit | 99.0% | 86.6% | (11) |
| Singleplex IHC – ALK | IHC EML4-ALK Fusion Testing | 100% | 99.0% | (12) |
| NGS testing | UW-OncoPlex | 99.2% | 100% | (13) |

Table S3. Medicines included in the model and assumed use within each type of treatment by scenario.

| Type of treatment | Medicine | Clinical trial | Scenario 1: Assumed use by type of treatment | Scenario 2: Assumed use by type of treatment | Scenario 3: Assumed use by type of treatment |
| --- | --- | --- | --- | --- | --- |
| Chemotherapy | Platinum doublet | Control arm of KEYNOTE-189 | 50% | 50% | 0% |
|  | Platinum doublet | Control arm of CheckMate 9LA | 50% | 50% | 0% |
| Immunotherapy - PD-L1 high | Pembrolizumab | KEYNOTE-024 | 0% | 0% | 33.3% |
|  | Cemiplimab | EMPOWER-Lung 1 | 0% | 0% | 33.3% |
|  | Atezolizumab | IMpower110 | 0% | 0% | 33.3% |
| Immuno-chemotherapy -PD-L1 non-high | Pembrolizumab | KEYNOTE-189 | 0% | 0% | 33.3% |
|  | Atezolizumab | IMpower150 | 0% | 0% | 33.3% |
|  | Nivolumab & Ipilimumab | CheckMate 9LA | 0% | 0% | 33.3% |
| Targeted therapy - EGFR | Erlotinib | EURTAC, OPTIMAL | 0% | 33% | 0% |
|  | Gefitinib | IPASS | 0% | 33% | 0% |
|  | Afatinib | LUX-Lung 3 | 0% | 33% | 0% |
|  | Dacomitinib | ARCHER 1050 | 0% | 0% | 0% |
|  | Osimertinib | FLAURA | 0% | 0% | 100% |
| Targeted therapy - ALK | Crizotinib | PROFILE 1014 | 0% | 100% | 0% |
|  | Ceritinib | ASCEND-4 | 0% | 0% | 0% |
|  | Alectinib | ALEX | 0% | 0% | 33.3% |
|  | Brigatinib | ALTA-1L | 0% | 0% | 33.3% |
|  | Lorlatinib | CROWN | 0% | 0% | 33.3% |
| Targeted therapy - ROS1 | Crizotinib | PROFILE 1001 | 0% | 0% | 50% |
|  | Entrectinib | ALKA-372-001, STARTRK-1, STARTRK-2 | 0% | 0% | 50% |
| Targeted therapy - BRAF | Dabrafenib & Trametinib | NCT01336634 | 0% | 0% | 100% |
| Targeted therapy - NTRK | Larotrectinib | NCT02122913, NCT02637687, NCT02576431 | 0% | 0% | 50% |
|  | Entrectinib | STARTRK-1, STARTRK-2, ALKA-372-001 | 0% | 0% | 50% |
| Targeted therapy - RET | Selpercatinib | LIBRETTO-001 | 0% | 0% | 50% |
|  | Pralsetinib | ARROW | 0% | 0% | 50% |
| Targeted therapy - MET | Capmatinib | GEOMETRY mono-1 | 0% | 0% | 50% |
|  | Tepotinib | VISION | 0% | 0% | 50% |

Notes: The platinum doublet contains a mix of carboplatin, cisplatin, pemetrexed, and paclitaxel.

Table S4. Administration-related inputs of included medicines in the model.

| Type of treatment | Medicine | Type of administration | Dosing | Treatment length |
| --- | --- | --- | --- | --- |
| Chemotherapy | Carboplatin | IV | AUC 5–6 mg/mL-min | 2–5 cycles |
|  | Cisplatin | IV | 75 mg/m² q3w | 4–5 cycles |
|  | Paclitaxel | IV | 200 mg/m² q3w | 2–5 cycles |
|  | Pemetrexed | IV | 500 mg/m² q3w | UDPoUT^ |
|  | Bevacizumab* | IV | 15 mg/kg q3w | UDPoUT |
| Immunotherapy | Pembrolizumab | IV | 200 mg q3w | UDPoUT or max. 2y |
|  | Cemiplimab | IV | 350 mg q3w | UDPoUT |
|  | Atezolizumab | IV | 1200 mg q3w | UDPoUT |
|  | Nivolumab | IV | 360 mg q3w | UDPoUT or max. 2y |
|  | Ipilimumab | IV | 1 mg/kg q6w | UDPoUT or max. 2y |
| EGFR | Erlotinib | Oral | 150 mg qDay | UDPoUT |
|  | Gefitinib | Oral | 250 mg qDay | UDPoUT |
|  | Afatinib | Oral | 40 mg qDay | UDPoUT |
|  | Dacomitinib | Oral | 45 mg qDay | UDPoUT |
|  | Osimertinib | Oral | 80 mg qDay | UDPoUT |
| ALK | Crizotinib | Oral | 250 mg BID | UDPoUT |
|  | Ceritinib | Oral | 450 mg qDay | UDPoUT |
|  | Alectinib | Oral | 600 mg BID | UDPoUT |
|  | Brigatinib | Oral | 180 mg qDay | UDPoUT |
|  | Lorlatinib | Oral | 100 mg qDay | UDPoUT |
| ROS1 | Crizotinib | Oral | 250 mg BID | UDPoUT |
|  | Entrectinib | Oral | 600 mg qDay | UDPoUT |
| BRAF | Dabrafenib | Oral | 150 mg BID | UDPoUT |
|  | Trametinib | Oral | 2 mg qDay | UDPoUT |
| NTRK | Larotrectinib | Oral | 100 mg BID | UDPoUT |
|  | Entrectinib | Oral | 600 mg qDay | UDPoUT |
| RET | Selpercatinib | Oral | 160 mg BID | UDPoUT |
|  | Pralsetinib | Oral | 400 mg qDay | UDPoUT |
| MET | Capmatinib | Oral | 400 mg BID | UDPoUT |
|  | Tepotinib | Oral | 450 mg qDay | UDPoUT |

Notes: AUC = area under the curve. UDPoUT = Until disease progression or unacceptable toxicity. IV = intravenous, qDay = once a day, BID = twice a day, mg = milligram, q3w = every three weeks, y = years. A body surface of 1.9 m², a body weight of 75 kg, and a serum creatinine level of 0.9 mg/dL were assumed in all countries. The dosing of carboplatin varied in the model according to the underlying clinical trial used in the analysis. The treatment length with carboplatin, cisplatin, and paclitaxel was four cycles in the ‘no testing’ and ‘sequential testing’ scenarios, whereas in the ‘multigene testing’ scenario the number of cycles followed the ones in the clinical trials of the combinations with immunotherapy. ^When pemetrexed was given in combination with pembrolizumab in the model a maximum treatment duration of two years was assumed, whereas when pemetrexed is given in combination with nivolumab in the model a maximum treatment duration of two three-weekly cycles was assumed. *Bevacizumab was only included in combination with atezolizumab in the model. Source: Medscape (<https://reference.medscape.com/>) for immunotherapy and targeted medicines, and control arms of pivotal clinical trials of immunotherapy for chemotherapy medicines (see Table S3).

Table S5. Monthly probability of treatment-related adverse events.

| Type of treatment | Medicine | Source / Assumption* | Anemia | Neutropenia | Fatigue | Nausea | Vomiting | Diarrhea | ASAT increase | ALAT increase |
| --- | --- | --- | --- | --- | --- | --- | --- | --- | --- | --- |
| Chemotherapy | Platinum doublet | Same as control arm of CheckMate 9LA | 6.38% | 3.90% | 0.11% | 0.34% | 0.58% | 0.23% | 0.00% | 0.00% |
|  | Platinum doublet | Control arm of CheckMate 9LA | 6.38% | 3.90% | 0.11% | 0.34% | 0.58% | 0.23% | 0.00% | 0.00% |
| Immunotherapy - PD-L1 high | Pembrolizumab | KEYNOTE-024 | 0.28% | 0.00% | 0.19% | 0.00% | 0.09% | 0.57% | 0.00% | 0.00% |
|  | Cemiplimab | EMPOWER-Lung 1 | 0.54% | 0.27% | 0.18% | 0.00% | 0.00% | 0.04% | 0.36% | 0.22% |
|  | Atezolizumab | IMpower110 | 0.33% | 0.13% | 0.13% | 0.07% | 0.07% | 0.00% | 0.33% | 0.33% |
| Immuno-chemotherapy -PD-L1 non-high | Pembrolizumab | Same as CheckMate 9LA | 0.99% | 1.13% | 0.37% | 0.23% | 0.28% | 0.65% | 0.00% | 0.23% |
|  | Atezolizumab | IMpower150 | 0.41% | 0.96% | 0.22% | 0.25% | 0.10% | 0.18% | 0.00% | 0.00% |
|  | Nivolumab & Ipilimumab | CheckMate 9LA | 0.99% | 1.13% | 0.37% | 0.23% | 0.28% | 0.65% | 0.00% | 0.23% |
| Targeted therapy - EGFR | Erlotinib | Same as LUX-Lung 3 | 0.04% | 0.04% | 0.12% | 0.08% | 0.28% | 1.40% | 0.00% | 0.00% |
|  | Gefitinib | Same as LUX-Lung 3 | 0.04% | 0.04% | 0.12% | 0.08% | 0.28% | 1.40% | 0.00% | 0.00% |
|  | Afatinib | LUX-Lung 3 | 0.04% | 0.04% | 0.12% | 0.08% | 0.28% | 1.40% | 0.00% | 0.00% |
|  | Dacomitinib | Same as LUX-Lung 3 | 0.09% | 0.00% | 0.00% | 0.00% | 0.00% | 0.57% | 0.03% | 0.06% |
|  | Osimertinib | FLAURA | 0.00% | 0.00% | 0.00% | 0.00% | 0.00% | 0.77% | 0.16% | 0.13% |
| Targeted therapy - ALK | Crizotinib | PROFILE 1014 | 0.06% | 0.50% | 0.00% | 0.31% | 0.31% | 0.19% | 1.03% | 1.59% |
|  | Ceritinib | Same as ALTA-1L | 0.00% | 0.00% | 0.00% | 0.16% | 0.08% | 0.16% | 0.16% | 0.16% |
|  | Alectinib | Same as ALTA-1L | 0.00% | 0.00% | 0.00% | 0.16% | 0.08% | 0.16% | 0.16% | 0.16% |
|  | Brigatinib | ALTA-1L | 0.00% | 0.00% | 0.00% | 0.16% | 0.08% | 0.16% | 0.16% | 0.16% |
|  | Lorlatinib | Same as ALTA-1L | 0.00% | 0.00% | 0.00% | 0.16% | 0.08% | 0.16% | 0.16% | 0.16% |
| Targeted therapy - ROS1 | Crizotinib | PROFILE 1001 | 0.00% | 0.16% | 0.00% | 0.00% | 0.03% | 0.00% | 0.03% | 0.06% |
|  | Entrectinib | ALKA-372-001, STARTRK-1, STARTRK-2 | 0.04% | 0.18% | 0.04% | 0.09% | 0.09% | 0.27% | 0.22% | 0.32% |
| Targeted therapy - BRAF | Dabrafenib & Trametinib | Same as LUX-Lung 3 | 0.00% | 0.00% | 0.00% | 0.16% | 0.08% | 0.16% | 0.16% | 0.16% |
| Targeted therapy - NTRK | Larotrectinib | NCT02122913, NCT02637687, NCT02576431 | 0.43% | 0.00% | 0.10% | 0.03% | 0.03% | 0.05% | 0.11% | 0.15% |
|  | Entrectinib | STARTRK-1, STARTRK-2, ALKA-372-001 | 1.58% | 0.38% | 0.97% | 0.00% | 0.00% | 0.19% | 0.19% | 0.19% |
| Targeted therapy - RET | Selpercatinib | LIBRETTO-001 | 0.00% | 0.00% | 0.00% | 0.00% | 0.05% | 0.10% | 0.41% | 0.68% |
|  | Pralsetinib | ARROW | 1.33% | 2.49% | 0.00% | 0.00% | 0.00% | 0.05% | 0.32% | 0.27% |
| Targeted therapy - MET | Capmatinib | GEOMETRY mono-1 | 0.00% | 0.00% | 0.18% | 0.11% | 0.13% | 0.02% | 0.16% | 0.37% |
|  | Tepotinib | VISION | 0.00% | 0.00% | 0.10% | 0.10% | 0.00% | 0.10% | 0.29% | 0.39% |

Notes: * Some clinical trials only reported total adverse events and not only treatment-related adverse events, in which case assumptions were made based on medicines in the same class of medicines (if possible).

Table S6. Unit costs for inputs sourced only for United States and converted with purchasing power parities to other countries (in 2021 US-dollars).

|  | Costs | Source |
| --- | --- | --- |
| Test costs |  |  |
| - EGFR test | 652 | (14) |
| - ALK test | 905 | (14) |
| - NGS test | 3,750 | (14) |
| - PD-L1 test | 924 | (14) |
| Monthly medical service use |  |  |
| - Chemotherapy | 6,352 | (15) |
| - Immunotherapy | 4,235 | (15) |
| - Targeted therapy | 1,791 | (15) |
| Treatment-related adverse events |  |  |
| - Anemia | 7,742 | (15) |
| - Neutropenia | 14,926 | (15) |
| - Fatigue | 491 | (16) |
| - Nausea | 79 | (17) |
| - Vomiting | 79 | (17) |
| - Diarrhea | 8,405 | (15) |
| - ASAT increase | 79 | (17) |
| - ALAT increase | 79 | (17) |

Notes: For the four adverse events nausea, vomiting, ASAT and ALAT increase, one outpatient visit for adverse events management was assumed.

Table S7. Country-specific unit costs (2021 US-dollars).

|  | Biopsy | Source | Monthly costs of chemotherapy medicines* | Monthly cost of immunotherapy medicines* | Monthly cost of targeted medicines* | Intravenous administration costs | Source | Monthly end-of-life care cost | Source | Monthly sick leave payments | Monthly disability pension payments | Source |
| --- | --- | --- | --- | --- | --- | --- | --- | --- | --- | --- | --- | --- |
| United States | 552 | (15) | 1–9,857 | 8,513–14,978 | 1,260–43,733 | 311 | (18) | 14,244 | (19) | 0 | 1,287 | (20-26) |
| Brazil | 342 | (27) | 19–1,449 | 406–1,990 | 602–10,495 | 121 | (28) | 1,100 | (29) | 243 | 867 | (20-26) |
| Germany | 110 | EBM code 13662 | 11–1,327 | 327–1,882 | 244–12,937 | 408 | (30) | 9,706 | (31) | 2,774 | 390 | (20-26) |
| Poland | 317 | assumption | 3–396 | 318–2,174 | 233–5,762 | 61 | (32) | 4,159 | (33) | 749 | 339 | (20-26) |
| Turkey | 80 | (34) | 2–721 | 362–3,003 | 206–5,635 | 50 | (35) | 3,179 | (36) | 403 | 133 | (20-26) |
| South Africa | 317 | assumption | 3–1,005 | 338–1,651 | 749–4,694 | 61 | assumption | 2,629 | assumption | 152 | 118 | (20-26) |
| China | 292 | (37) | 1–938 | 471–2,294 | 64–2,643 | 2 | (38) | 7,023 | (39) | 517 | 326 | (20-26) |
| Japan | 383 | (40) | 6–455 | 913–3,252 | 406–12,822 | 14 | (41) | 8,265 | (42) | 1,648 | 1,489 | (20-26) |
| Australia | 151 | MBS item 38812 | 1–361 | 448–2,016 | 606–7,883 | 78 | MBS item 13950 | 4,058 | (43) | 883 | 381 | (20-26) |

Notes: Unit costs for a biopsy in Poland and South Africa were assumed to be equal to the mean costs in Brazil and China. Unit costs for intravenous administration and for end-of-life care in South Africa were assumed to be equal to the mean costs in Brazil, China, and Poland. The monthly sick leave payments shown here have been multiplied with the national employment rates, and possible waiting times for payments to set in have not been taken into account. * The three classes of medicines follow the classification in Table S4, and the range of the individual monthly costs (based on list prices from Eversana) of the included medicines is shown.

Table S8. Sensitivity analysis of sensitivity and specificity of biomarker tests for main outcomes per patient and a 5-year time horizon.

|  |  | No testing | | Sequential testing | | Multigene testing | |
| --- | --- | --- | --- | --- | --- | --- | --- |
|  |  | Life years | Total costs (in USD) | Life years | Total costs (in USD) | Life years | Total costs (in USD) |
| United States | Scenario 1 | 1.36 | 153,044 | 1.67 | 183,158 | 2.26 | 398,625 |
|  | Base case | 1.36 | 153,044 | 1.73 | 188,582 | 2.27 | 401,313 |
|  | Scenario 2 | 1.36 | 76,318 | 1.86 | 97,335 | 2.10 | 600,313 |
| Brazil | Scenario 1 | 1.36 | 76,318 | 1.68 | 87,683 | 2.25 | 140,736 |
|  | Base case | 1.36 | 76,318 | 1.73 | 89,673 | 2.26 | 141,976 |
|  | Scenario 2 | 1.36 | 76,318 | 1.86 | 97,335 | 2.13 | 203,579 |
| Germany | Scenario 1 | 1.36 | 120,032 | 1.60 | 128,239 | 2.15 | 180,174 |
|  | Base case | 1.36 | 120,032 | 1.66 | 129,917 | 2.16 | 181,102 |
|  | Scenario 2 | 1.36 | 120,032 | 1.80 | 137,808 | 2.00 | 221,194 |
| Poland | Scenario 1 | 1.36 | 62,416 | 1.60 | 69,522 | 2.15 | 107,825 |
|  | Base case | 1.36 | 62,416 | 1.66 | 71,071 | 2.16 | 108,660 |
|  | Scenario 2 | 1.36 | 62,416 | 1.80 | 78,169 | 2.00 | 161,862 |
| Turkey | Scenario 1 | 1.36 | 44,144 | 1.60 | 50,264 | 2.19 | 92,203 |
|  | Base case | 1.36 | 44,144 | 1.66 | 51,628 | 2.20 | 92,993 |
|  | Scenario 2 | 1.36 | 44,144 | 1.80 | 57,860 | 1.95 | 137,749 |
| South Africa | Scenario 1 | 1.36 | 64,069 | 1.63 | 71,429 | 2.22 | 104,595 |
|  | Base case | 1.36 | 64,069 | 1.68 | 72,892 | 2.23 | 105,271 |
|  | Scenario 2 | 1.36 | 64,069 | 1.82 | 78,515 | 1.98 | 107,837 |
| China | Scenario 1 | 1.36 | 82,140 | 1.87 | 89,234 | 2.56 | 120,130 |
|  | Base case | 1.36 | 82,140 | 1.92 | 89,953 | 2.59 | 120,388 |
|  | Scenario 2 | 1.36 | 82,140 | 2.00 | 92,615 | 2.31 | 74,748 |
| Japan | Scenario 1 | 1.36 | 121,696 | 1.76 | 138,937 | 2.40 | 207,141 |
|  | Base case | 1.36 | 121,696 | 1.81 | 141,205 | 2.42 | 208,944 |
|  | Scenario 2 | 1.36 | 121,696 | 1.92 | 148,656 | 2.18 | 279,068 |
| Australia | Scenario 1 | 1.36 | 133,071 | 1.60 | 143,708 | 2.15 | 196,732 |
|  | Base case | 1.36 | 133,071 | 1.66 | 145,892 | 2.16 | 197,746 |
|  | Scenario 2 | 1.36 | 133,071 | 1.80 | 155,413 | 2.00 | 223,235 |

Notes: Scenario 1 uses the upper bound of the 95% confidence interval (CI) of the specificity of different tests and the lower bound of the 95% CI of the sensitivity of the tests (see Table S2). Scenario 2 uses the lower bound of the 95% CI of the specificity of different tests and the upper bound of the 95% CI of the sensitivity of the tests. For the ALK test, the sensitivity and specificity were varied by +/-10% (restricting the upper bound to 100%) in absence of a published 95% CI.

# References

1. Dietel M, Savelov N, Salanova R, Micke P, Bigras G, Hida T, et al. *Real-world prevalence of programmed death ligand 1 expression in locally advanced or metastatic non-small-cell lung cancer: The global, multicenter EXPRESS study*. Lung Cancer. 2019;134:174-9.

2. Si X, Pan R, Ma S, Li L, Liang L, Zhang P, et al. *Genomic characteristics of driver genes in Chinese patients with non-small cell lung cancer*. Thorac Cancer. 2021;12(3):357-63.

3. Zhang YL, Yuan JQ, Wang KF, Fu XH, Han XR, Threapleton D, et al. *The prevalence of EGFR mutation in patients with non-small cell lung cancer: a systematic review and meta-analysis*. Oncotarget. 2016;7(48):78985-93.

4. Chia PL, Mitchell P, Dobrovic A, John T. *Prevalence and natural history of ALK positive non-small-cell lung cancer and the clinical impact of targeted therapy with ALK inhibitors*. Clin Epidemiol. 2014;6:423-32.

5. Gainor JF, Shaw AT. *Novel targets in non-small cell lung cancer: ROS1 and RET fusions*. Oncologist. 2013;18(7):865-75.

6. Alvarez JGB, Otterson GA. *Agents to treat BRAF-mutant lung cancer*. Drugs Context. 2019;8:212566.

7. Leonetti A, Facchinetti F, Rossi G, Minari R, Conti A, Friboulet L, et al. *BRAF in non-small cell lung cancer (NSCLC): Pickaxing another brick in the wall*. Cancer Treat Rev. 2018;66:82-94.

8. Liu F, Wei Y, Zhang H, Jiang J, Zhang P, Chu Q. *NTRK Fusion in Non-Small Cell Lung Cancer: Diagnosis, Therapy, and TRK Inhibitor Resistance*. Front Oncol. 2022;12:864666.

9. Cascetta P, Sforza V, Manzo A, Carillio G, Palumbo G, Esposito G, et al. *RET Inhibitors in Non-Small-Cell Lung Cancer*. Cancers (Basel). 2021;13(17).

10. Lee JK, Madison R, Classon A, Gjoerup O, Rosenzweig M, Frampton GM, et al. *Characterization of Non-Small-Cell Lung Cancers With MET Exon 14 Skipping Alterations Detected in Tissue or Liquid: Clinicogenomics and Real-World Treatment Patterns*. JCO Precis Oncol. 2021;5.

11. US FDA. Summary of safety and effectiveness data (SSED). Available from: <https://www.accessdata.fda.gov/cdrh_docs/pdf12/P120022B.pdf> [accessed May 12, 2022].

12. Djalalov S, Beca J, Hoch JS, Krahn M, Tsao MS, Cutz JC, et al. *Cost effectiveness of EML4-ALK fusion testing and first-line crizotinib treatment for patients with advanced ALK-positive non-small-cell lung cancer*. J Clin Oncol. 2014;32(10):1012-9.

13. Pritchard CC, Salipante SJ, Koehler K, Smith C, Scroggins S, Wood B, et al. *Validation and implementation of targeted capture and sequencing for the detection of actionable mutation, copy number variation, and gene rearrangement in clinical cancer specimens*. J Mol Diagn. 2014;16(1):56-67.

14. Vanderpoel J, Stevens AL, Emond B, Lafeuille MH, Hilts A, Lefebvre P, et al. *Total cost of testing for genomic alterations associated with next-generation sequencing versus polymerase chain reaction testing strategies among patients with metastatic non-small cell lung cancer*. J Med Econ. 2022;25(1):457-68.

15. Harvey MJ, Cunningham R, Sawchyn B, Montesion M, Reddy P, McBride A, et al. *Budget Impact Analysis of Comprehensive Genomic Profiling in Patients With Advanced Non-Small-Cell Lung Cancer*. JCO Precis Oncol. 2021;5:1611-24.

16. Qiao N, Insinga R, Burke T, Lopes G. *Cost-Minimization Analysis of Pembrolizumab Monotherapy Versus Nivolumab in Combination with Ipilimumab as First-Line Treatment for Metastatic PD-L1-Positive Non-small Cell Lung Cancer: A US Payer Perspective*. Pharmacoecon Open. 2021;5(4):765-78.

17. Huang M, Lopes GL, Insinga RP, Burke T, Ejzykowicz F, Zhang Y, et al. *Cost-effectiveness of pembrolizumab versus chemotherapy as first-line treatment in PD-L1-positive advanced non-small-cell lung cancer in the USA*. Immunotherapy. 2019;11(17):1463-78.

18. Johnson & Johnson Health Care Systems. 2021 Final Medicare Coding & Payment for Drug Administration Services Under the Hospital Outpatient Prospective Payment System. Available from: <https://www.janssencarepath.com/sites/www.janssencarepath-v1.com/files/medicare-coding-and-payment-drug-admin-services-hospital-outpatient.pdf> [accessed Jul 12, 2022].

19. Bremner KE, Krahn MD, Warren JL, Hoch JS, Barrett MJ, Liu N, et al. *An international comparison of costs of end-of-life care for advanced lung cancer patients using health administrative data*. Palliat Med. 2015;29(10):918-28.

20. Eurostat. Pensions beneficiaries at 31st December. Available from: <https://ec.europa.eu/eurostat/databrowser/explore/all/all_themes>.

21. Eurostat. Tables by benefits - disability function. Available from: <https://ec.europa.eu/eurostat/databrowser/explore/all/all_themes> [accessed Apr 7, 2022].

22. International Labour Organization. Country Profiles. Available from: <https://ilostat.ilo.org/data/country-profiles/> [accessed Apr 7, 2022].

23. International Social Security Association. Country profiles. Available from: <https://ww1.issa.int/country-profiles> [accessed Apr 7, 2022].

24. Japan Pension Service. National Pension System. Available from: <https://www.nenkin.go.jp/international/japanese-system/nationalpension/nationalpension.html> [accessed Apr 7, 2022].

25. Social Security Administration. Disabled worker average benefits. Available from: <https://www.ssa.gov/oact/STATS/dib-g3.html> [accessed Apr 7, 2022].

26. World Bank. DataBank - World Development Indicators. Available from: <https://databank.worldbank.org/source/world-development-indicators> [accessed May 4, 2022].

27. Santos M, Custodio MG, Matsuo AL, Montenegro G, Pepe C, Asano E, et al. *Cost effectiveness analysis of plasma genotyping versus tumor genotyping in detection of advanced non-small-cell lung cancer with epidermal growth factor receptor and T790M mutation under the Brazilian private healthcare system perspective*. J Bras Econ Saúde. 2018;10(3):262-8.

28. Piha T, Marques M, Paladini L, Teich V. *Cost-effectiveness analysis of gefitinib versus chemotherapy protocols in the first line treatment of non small-cell EGFR positive lung cancer*. J Bras Econ Saúde. 2011;3(3):269-77.

29. Lana AP, Perelman J, Gurgel Andrade EI, Acurcio F, Guerra AA, Jr., Cherchiglia ML. *Cost Analysis of Cancer in Brazil: A Population-Based Study of Patients Treated by Public Health System From 2001-2015*. Value Health Reg Issues. 2020;23:137-47.

30. Schremser K, Rogowski WH, Adler-Reichel S, Tufman AL, Huber RM, Stollenwerk B. *Cost-Effectiveness of an Individualized First-Line Treatment Strategy Offering Erlotinib Based on EGFR Mutation Testing in Advanced Lung Adenocarcinoma Patients in Germany*. Pharmacoeconomics. 2015;33(11):1215-28.

31. Andreas S, Chouaid C, Danson S, Siakpere O, Benjamin L, Ehness R, et al. *Economic burden of resected (stage IB-IIIA) non-small cell lung cancer in France, Germany and the United Kingdom: A retrospective observational study (LuCaBIS)*. Lung Cancer. 2018;124:298-309.

32. Tilden D, Aristides M, Stynes G, Orlewska E, Krzakowski M, Jassem J, et al. *Cost-effectiveness of gemcitabine in combination with cisplatin versus vinorelbine in combination with cisplatin in the treatment of non-small cell lung cancer in Poland: a retrospective economic analysis of clinical trials*. Farmakoekonomika. 2003;3.

33. Cialkowska-Rysz AD, Pokropska W, Luczak J, Kaptacz A, Stachowiak A, Hurich K, et al. *How much does care in palliative care wards cost in Poland?* Arch Med Sci. 2016;12(2):457-68.

34. Cicin I, Oksuz E, Karadurmus N, Malhan S, Gumus M, Yilmaz U, et al. *Economic burden of lung cancer in Turkey: a cost of illness study from payer perspective*. Health Econ Rev. 2021;11(1):22.

35. Öksüz E, Malhan S, Yanik L, Koc E, Erdogan-Ciftci E, Guler B, et al. *Cost-effectiveness of Alectinib in the First-Line Treatment of Advanced ALK-Positive Non-Small Cell Lung Cancer in Turkey*. Value in Health. 2018;21:S40.

36. Saygili M, Celik Y. *An evaluation of the cost-effectiveness of the different palliative care models available to cancer patients in Turkey*. Eur J Cancer Care (Engl). 2019;28(5):e13110.

37. Wu B, Gu X, Zhang Q. *Cost-Effectiveness of Osimertinib for EGFR Mutation-Positive Non-Small Cell Lung Cancer after Progression following First-Line EGFR TKI Therapy*. J Thorac Oncol. 2018;13(2):184-93.

38. Rui M, Fei Z, Wang Y, Zhang X, Ma A, Sun H, et al. *Cost-effectiveness analysis of sintilimab + chemotherapy versus camrelizumab + chemotherapy for the treatment of first-line locally advanced or metastatic nonsquamous NSCLC in China*. J Med Econ. 2022;25(1):618-29.

39. Zeng X, Karnon J, Wang S, Wu B, Wan X, Peng L. *The cost of treating advanced non-small cell lung cancer: estimates from the chinese experience*. PLoS One. 2012;7(10):e48323.

40. Osada H, Kojima K, Tsukada H, Nakajima Y, Imamura K, Matsumoto J. *Cost-effectiveness associated with the diagnosis and staging of non-small-cell lung cancer*. Jpn J Thorac Cardiovasc Surg. 2001;49(1):1-10.

41. Narita Y, Matsushima Y, Shiroiwa T, Chiba K, Nakanishi Y, Kurokawa T, et al. *Cost-effectiveness analysis of EGFR mutation testing and gefitinib as first-line therapy for non-small cell lung cancer*. Lung Cancer. 2015;90(1):71-7.

42. Awano N, Izumo T, Inomata M, Kuse N, Tone M, Takada K, et al. *Medical costs of Japanese lung cancer patients during end-of-life care*. Jpn J Clin Oncol. 2021;51(5):769-77.

43. Goldsbury DE, Yap S, Weber MF, Veerman L, Rankin N, Banks E, et al. *Health services costs for cancer care in Australia: Estimates from the 45 and Up Study*. PLoS One. 2018;13(7):e0201552.
